# Newspapers

**Published:** 1857-02

**Authors:** 


### NEWSPAPERS.

It may occasionally require some scrutiny to discover what possible connection there can be between some of our articles and the more direct object of our journal; but if our readers will wait until the close of our articles, we will do what sometimes railroad conductors, for the benefit of hotel-keepers, fail to do *on purpose;* that is, "make the connection." So with health and newspapers, we concatenate them as follows:

If you make your child religious, you keep him out of the street without an effort; for he has no desire to mingle with its associations, either in the day time or at night. One of the first steps towards making a child religious, is to *make home inviting* by affection, and sympathy, and pleasurable employment. In such an atmosphere we believe any youth will grow up safe in practice and in principle. The young *will* be employed. It is the business and the duty of a parent to see to it, that the employment is useful, agreeable, enticing. The reading of newspapers regularly may be made to combine these results. But these newspapers must be of a proper character, and of such there are very few. It requires no slight power of discrimination to make a safe selection. Without specifying by name that large number *not fit to be opened around the family fireside*, by reason of their inane love stories, their indecent advertisements, their slang phrases, their profane blanks, and stars, and initials, their whole columns of bigamies, abductions, seductions, rapes, and the like; by reason, more especially, of their frequent side-flings against professing christians, clergymen, the church, and religion, to say nothing of the uncourteous language, violent personal abuse, and low epithets, which so often blur the columns of partisan newspapers, secular and religious, we say, without any invidious specifications, we urge upon heads of families the duty and advantage of providing suitable newspaper reading, excluding every one, however unexceptionable as a general rule, the moment it offends in any of the above-named directions. For the better the paper is uniformly, the more pernicious is the influence of an occasional dereliction as to the points named.

